# Mesenteric malperfusion syndrome is the game changer in acute aortic dissection

**DOI:** 10.1016/j.xjon.2024.03.014

**Published:** 2024-03-30

**Authors:** Koray Ak

**Affiliations:** Department of Cardiovascular Surgery, Marmara University Faculty of Medicine, İstanbul, Turkey

To the Editor:

**Figure:**
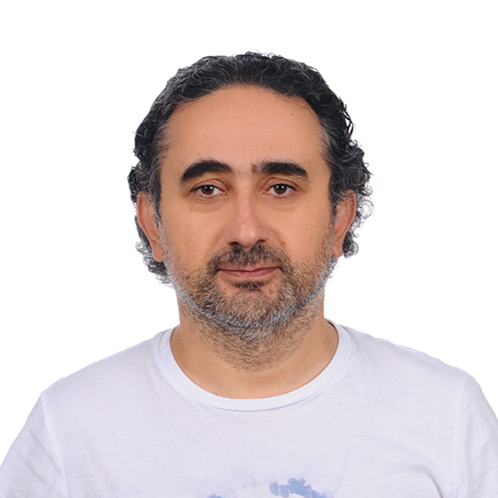

I read with great interest the article by Brown and colleagues.^1^ They stated that malperfusion syndrome (MPS) increases the risk for in-hospital mortality and that the number of malperfused vascular beds has critical importance in defining the risk of mortality after acute type A aortic dissection repair. The authors must be congratulated for their successful outcome. I have several concerns about the study.

I concur with the authors' findings that rapid aortic repair successfully restores most patients' true lumen perfusion in MPS. Also, I support the outcome that having more than 1 malperfused vascular bed results in a worse prognosis after central repair. However, it has been shown that mesenteric MPS (mes-MPS) is a separate subgroup and some of these patients do not benefit from the aortic repair first policy, which was shown to be associated with more than 80% early mortality.^2^ Because the coronary, supra-aortic, and iliofemoral vessels are in direct surgical view of the surgeon, it is technically easier to establish true lumen perfusion during central repair in case of MPS. On the contrary, the mesenteric circulation is rather a hidden zone and the surgeon has to take individualized decisions to improve the outcome of this subgroup of patients. In this regard, the severity of mes-MPS gains priority in the decision-making process preoperatively (rapid vs delayed aortic repair). The authors reported that of the 135 patients with MPS, 13 had visceral MPS and 26 had renal MPS. They did not mention the details related to the severity of preoperative mes-MPS, such as biochemistry, lactate level, presence or absence of abdominal pain, and ischemic colitis with bloody diarrhea. Putting the patients with a slightly elevated lactate level related to mild mes-MPS and those who had severe mes-MPS with bloody diarrhea and acidosis into the same basket could veil the impact of mes-MPS on the early outcome after aortic repair. Furthermore, deepening metabolic acidosis and hyperlactatemia related to severe mes-MPS throughout the procedure would make the weaning from cardiopulmonary bypass almost impossible in most cases.^3^ Overall, the severity of mes-MPS should have been taken into consideration in the determination of rapid aortic repair or mesenteric revascularization should be performed initially in acute type A aortic dissection.

## Conflict of Interest Statement

The author reported no conflicts of interest.

The *Journal* policy requires editors and reviewers to disclose conflicts of interest and to decline handling or reviewing manuscripts for which they may have a conflict of interest. The editors and reviewers of this article have no conflicts of interest.
